# First results of the Brazilian Registry of Percutaneous Left Atrial Appendage Closure

**DOI:** 10.5935/abc.20170150

**Published:** 2017-11

**Authors:** Ênio Eduardo Guérios, Francisco Chamié, Márcio Montenegro, Eduardo Benchimol Saad, Fabio Sandoli de Brito Junior, Paulo Avancini Caramori, Luiz Carlos Simões, Flávio Roberto Azevedo de Oliveira, Luiz Carlos Giuliano, Cláudio Munhoz da Fontoura Tavares

**Affiliations:** 1 Hospital Pilar, Curitiba, PR - Brasil; 2 Hospital dos Servidores do Estado do Rio de Janeiro, Rio de Janeiro, RJ - Brasil; 3 Instituto Estadual de Cardiologia Aloysio de Castro (IECAC), Rio de Janeiro, RJ - Brasil; 4 Hospital Pró-Cardíaco, Rio de Janeiro, RJ - Brasil; 5 Instituto Nacional de Cardiologia (INC - MS), Rio de Janeiro, RJ - Brasil; 6 Hospital São Vicente de Paulo, Rio de Janeiro, RJ - Brasil; 7 Rede D'Or, Rio de Janeiro, RJ - Brasil; 8 Hospital Israelita Albert Einstein, São Paulo, SP - Brasil; 9 Centro de Pesquisa Cardiovasculares do Hospital São Lucas da Pontifícia Universidade Católica do Rio Grande do Sul (PUCRS), Porto Alegre, RS - Brasil; 10 Instituto de Medicina Integral Prof. Fernando Figueira, Recife, PE - Brasil; 11 SOS Cardio Serviços Hospitalares, Florianópolis, SC - Brasil

**Keywords:** Atrial Fibrillation, Septal Occluder Devices, Atrial Appendage, Stroke, Cardiovascular Surgical Procedures, Medical Records

## Abstract

**Background:**

Left atrial appendage closure (LAAC) is an effective alternative to oral anticoagulation (OA) for the prevention of stroke in patients with non-valvular atrial fibrillation (NVAF).

**Objective:**

To present the immediate results and late outcomes of patients submitted to LAAC and included in the Brazilian Registry of Percutaneous Left Atrial Appendage Closure.

**Methods:**

91 patients with NVAF, high stroke risk (CHA_2_DS_2_VASc score = 4.5 ± 1.5) and restrictions to OAC (HAS-BLED score = 3.6 ± 1.0) underwent 92 LAAC procedures using either the Amplatzer cardiac plug or the Watchman device in 11 centers in Brazil, between late 2010 and mid 2016.

**Results:**

Ninety-six devices were used (1.04 device/procedure, including an additional non-dedicated device), with a procedural success rate of 97.8%. Associated procedures were performed in 8.7% of the patients. Complete LAAC was obtained in 93.3% of the successful cases. In cases of incomplete closure, no residual leak was larger than 2.5 mm. One patient needed simultaneous implantation of 2 devices. There were 7 periprocedural major (5 pericardial effusions requiring pericardiocentesis, 1 non-dedicated device embolization and 1 coronary air embolism without sequelae) and 4 minor complications. After 128.6 patient-years of follow-up there were 3 deaths unrelated to the procedure, 2 major bleedings (one of them in a patient with an unsuccessful LAAC), thrombus formation over the device in 2 cases (both resolved after resuming OAC for 3 months) and 2 strokes (2.2%).

**Conclusions:**

In this multicenter, real world registry, that included patients with NVAF and high thromboembolic and bleeding risks, LAAC effectively prevented stroke and bleeding when compared to the expected rates based on CHA_2_DS_2_VASc and HASBLED scores for this population. Complications rate of the procedure was acceptable considering the beginning of the learning curve of most of the involved operators.

## Introduction

Although still significantly underdiagnosed,^1^ atrial fibrillation (AF) is a public health issue with major socio-economic impact, and its relative incidence has constantly grown over the years.^2^ One of the greatest risks of this arrhythmia is left atrial thrombus formation, which occurs in 10% of patients with AF (even when acute), and it is associated with a 3.5 times elevated risk for stroke, reaching average annual rates of 5%.^3-5^ In order to prevent this devastating complication, the Guidelines recommend oral anticoagulation (OAC) with vitamin K antagonists or one of the new oral anticoagulants (NOACs) as Class I for the treatment of patients with non-valvular atrial fibrillation (NVAF) and at high risk for stroke, defined by the CHA_2_DS_2_-VASc score.^6^ In spite of being quite effective, these drugs depend on treatment adherence and, more importantly, their use is associated with high risk of bleeding.^7,8^

As a “local therapy” that does not depend on adherence and reduces the risk of bleeding, left atrial appendage closure (LAAC) proved to be an effective alternative to OAC for the prevention of stroke in patients with non-valvular atrial fibrillation (NVAF), with lower bleeding risk.^9^ In a recent meta-analysis, including about 88000 patients, LAAC has also shown to be superior to placebo and to double antiplatelet therapy and comparable to the NOACs in the prevention of mortality and stroke or systemic embolism in these patients, with a similar bleeding risk.^10^

In spite of its great therapeutic potential and a vertiginous growth of its indication and application in other countries, the LAAC procedure is still little known and little used in Brazil, with scarce data in the national literature. This article aims to report the results of the largest Brazilian multicenter registry of LAAC.

## Methods

Ninety-one consecutive patients with permanent or paroxysmal NVAF, with high stroke risk and restrictions to OAC, underwent 92 LAAC procedures between 2010 and 2016 in 11 Brazilian centers. All patients that underwent LAAC in these centers were included, and the data related to the procedures and to the follow-up of patients were collected prospectively and analyzed retrospectively.

**Figure 1:**
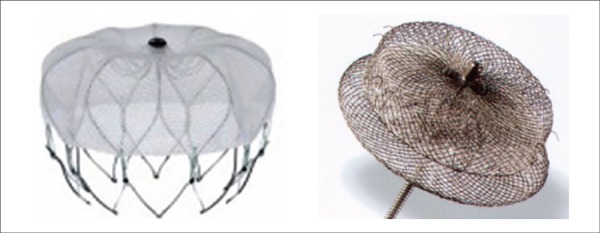
Watchman device (left) and Amplatzer Cardiac Plug (right)

A preoperative evaluation with transesophageal echocardiography (TEE) was performed in all patients. Patients with LAA thrombus or LAA anatomy deemed unfavorable to intervention (landing zone < 13 mm or > 30 mm or LA depth < 10 mm) were excluded. For the eligible patients, the OACs were suspended when in use, 3-5 days pre-procedure. All the interventions were guided simultaneously by angiography and intraoperative TEE, and one of the 2 devices available in the Brazilian market (Figure 1) was implanted: the Amplatzer Cardiac Plug (ACP, St. Jude Medical, St. Paul, MN), available since 2010, and the Watchman (Boston Scientific, Marlborough, MA), available since mid-2015. Both devices and their respective implant techniques have been described previously in detail.^9,11^

Procedural success was defined as effective implantation of the occluder device in the LAA, without periprosthetic residual flow larger than 5 mm, according to evaluation of the intraoperative TEE. Major adverse events were defined as the occurrence of death, stroke, systemic embolization, device embolization, acute myocardial infarction, pericardial effusion with cardiac tamponade or bleeding with the need for transfusion, data collected and reported during both hospitalization and follow-up.

The follow-up considered the practice of each investigator, but it included at least one clinical visit in every center and one control TEE carried out from three months after the procedure, searching for the detection and quantification of periprosthetic residual flow or thrombus formation over the prosthesis. In case there is no finding or adverse event, the last follow-up available was considered in the analysis.

### Statistical analysis

The statistical analysis was performed using the IBM SPSS Statistics v.20 software. Data for categorical variables were presented as frequencies and proportion. Continuous variables with normal distribution were described by mean ± standard deviation and compared through Student's t-test for paired samples. Other quantitative variables were described by median, first quartile and third quartile. The condition of normality was evaluated using the Kolmogorov-Smirnov test. Values of p < 0.05 were statistically significant.

## Results

**Figure 2:**
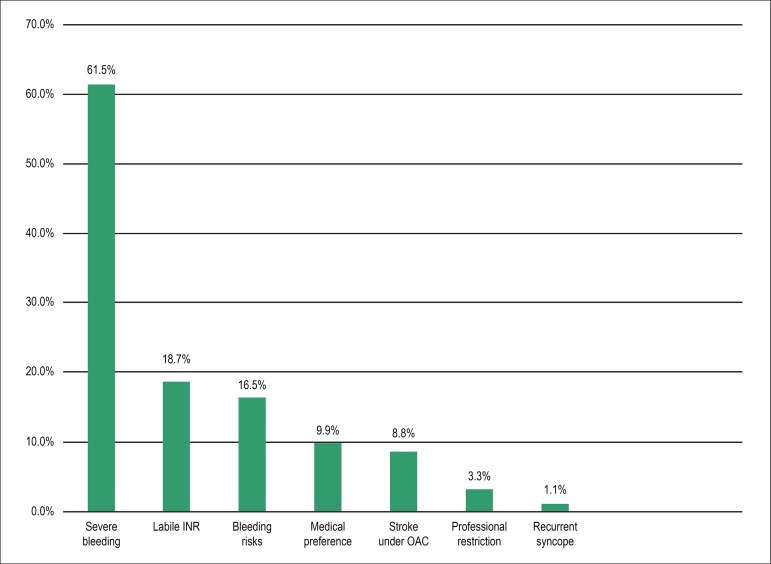
Contraindications to oral anticoagulation*. INR: international normalized ratio; OAC: oral anticoagulation. * the same patient may have multiple contraindications.

The clinical characteristics of patients are detailed in Table 1. Ninety-one patients (males 59.3%, mean age = 73.1 ± 10.1 years) with NVAF (62.6% permanent, 37.4% paroxysmal) and at high risk for systemic embolism (CHA_2_DS_2_-VASc score = 4.5 ± 1.5, 49.5% with previous stroke) and for bleeding (HAS-BLED score = 3.6 ± 1.0, 61.5% with previous bleeding episodes while on OAC - Table 2) were treated. Major indications for LAAC were important previous bleeding episodes (mainly gastrointestinal or neurological) or labile INR (Figure 2). Sixty-eight percent of patients were deemed ineligible for OAC by their clinicians, whether with vitamin K antagonists or one of the NOACs.

**Table 1:** Clinical Characteristics of Patients (n = 91)

Variable	Result*
Age (years)	73.1 ± 10.1
65-75	27 (29.7)
>75	47 (51.6)
Male	54 (59.3)
**Atrial Fibrillation**	
Permanent	57 (62.6)
Paroxysmal	34 (37.4)
LVEF (%)	58.2 ± 13.4
CHADS_2_score	3.1 ± 1.3
CHA_2_DS_2_-VASc score	4.5 ± 1.5
HAS-BLED score	3.6 ± 1.0
Ineligible for OA	62 (68.1)
Congestive cardiac failure	28 (30.8)
High blood pressure	78 (85.7)
Diabetes	33 (36.3)
Previous CVA	45 (49.5)
Peripheral vascular disease	22 (24.2)
Renal and liver dysfunction	21 (23.1)
Previous bleeding	56 (61.5)
Labil INR	27 (29.7)
Drugs or alcohol	21 (23.1)

* Mean ± standard deviation or frequency (percentage). LVEF: left ventricular ejection fraction; OA: oral anticoagulation; CVA: cerebrovascular accident; INR: international normalized ratio.

**Table 2:** patients distribution according to CHADS_2_, CHA_2_DS_2_-VASc and HAS-BLED scores (n = 91)

Score	CHADS_2_	CHA_2_DS_2_-VASc	HAS-BLED
	n (%)	n (%)	n (%)
0	0	0	0
1	10 (11.0)	1 (1.1)	1 (1.1)
2	21 (23.0)	9 (9.9)	14 (15.4)
3	27 (29.7)	13 (14.3)	27 (29.7)
4	18 (19.8)	26 (28.6)	32 (35.2)
5	12 (13.2)	19 (20.8)	16 (17.5)
6	3 (3.3)	15 (16.5)	1 (1.1)
7	n/a	6 (6.6)	0
8	n/a	1 (1.1)	n/a
9	n/a	1 (1.1)	n/a
mean ± SD	3.1 ± 1.3	4.5 ± 1.5	3.6 ± 1.0

SD: standard deviation; n/a: not applicable.

**Figure 3:**
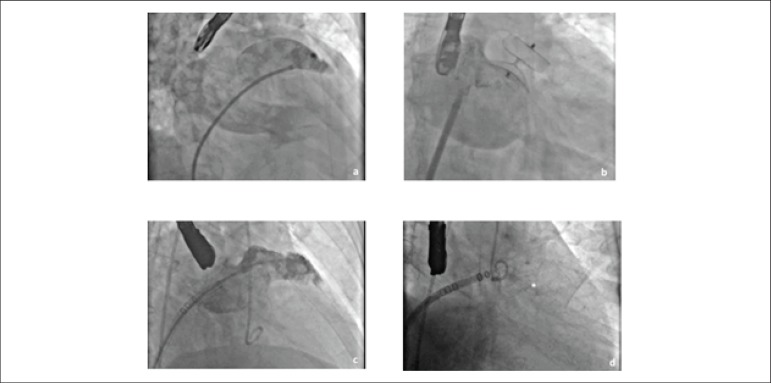
Implantation of the Amplatzer Cardiac Plug (ACP) and Watchman devices. 3a and 3c) left atrial appendage angiographies, pre-occlusion; 3b) Post-implantation, ACP device; 3d) Post-implantation, Watchman device (*).

Procedure-related data are presented in Table 3. Forty-five percent of the 92 interventions were performed with the aid of a proctor. An ACP was implanted in 94.6% of cases, and a Watchman device in 5.4% (Figure 3). A total of 96 occluder devices was used in 92 procedures (1.04 device/procedure). Prosthesis implantation was successful in 97.8% of cases. The procedure was aborted in two patients due to short LAA depth (< 10 mm) in one of the patients, and to an oversized landing zone (> 30 mm) in another, both characteristics underestimated in the initial TEE. Due to an incomplete closure of the LAA following the implantation of an ACP 16 mm, one patient received an additional non-dedicated device (a septal occluder of 25 mm in diameter), with good initial results. However, a control fluoroscopy performed after 4 days revealed embolization of the device to the aortic arch. The prosthesis was removed percutaneously, and a second ACP 28 mm was successfully implanted over the initial ACP 16 mm, which led to complete closure of the LAA.

**Table 3:** Periprocedural data (n = 92)

Variable	Result*
**Access**	
Transseptal	85 (92.4)
PFO/IC	7 (7.6)
**LAA diameter (implant zone)**	
Angiography (mm)	20.9 ± 4.2
TEE (mm)	20.4 ± 4.3
**Device oversizing**	
Angiography (%)	18.1 ± 9.1
TEE (%)	21.5 ± 13.0
**Implanted device (n)**	
ACP	87 (94.6)
Watchman	5 (5.4)
Non-dedicated device	1 (1.1)
Devices used per procedure	1.04
Sucess	90 (97.8)
Complete occlusion of the LAA	84 (93.3**)
**Associated intervention**	
PFO occlusion	4 ( 4.4)
IC occlusion	2 (2.2)
Coronary angioplasty	2 (2.2)
**Major adverse events**	
Procedure-related death	0
CVA	0
Coronary air embolism	1 (1.1)
TIA	0
Embolization of dedicated device	1 (1.1)
Acute myocardial infarction	0
**Cardiac tamponade**	
Acute	2 (2.2)
Late (> 24h)	3 (3.3)
Major bleeding	0

* Mean ± Standard deviation (percentage);

** Considering successful cases. PFO: patent foramen ovale; IC: interatrial communication; LAA: left atrial appendage; TEE: transesophageal echocardiography; ACP: Amplatzer Cardiac Plug; CVA: cerebrovascular accident; TIA: transient ischemic attack.

**Figure 4:**
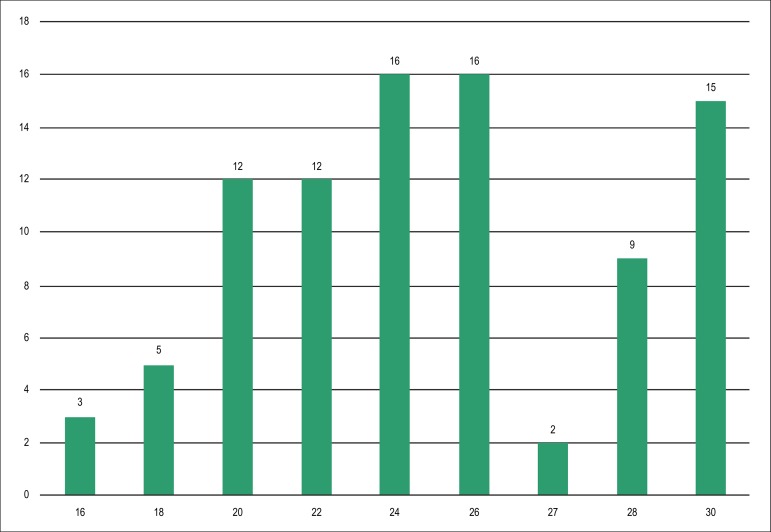
Distribution of the sizes of the implanted devices (mm). Data are expressed in number of devices

The average diameter of the implanted prosthesis was 24.2 ± 3.8 mm, corresponding to the mean left atrial appendage dimensions of 20.4 ± 4.3 mm derived from TEE and 20.9 ± 4.1 mm from angiography (p = 0.012 between the diagnostic methods). Thus, the average oversizing of the implanted device was 21.5 ± 13% based on the TEE measurement and 18.1 ± 9.1% according to the angiography. The prosthesis sizes most frequently used were 24 and 26 mm (Figure 4), and the first selected device was effectively implanted in 95.6% of successful cases. Concomitant procedures (coronary angioplasty, closure of an atrial septal defect or patent foramen ovale) were performed along with LAAC in 8.7% of cases. Average fluoroscopy time was 16.7 ± 8.7 minutes and a mean contrast volume of 157.5 ± 81.8 ml was used per procedure. The absence of periprosthetic residual flow was verified in 93.3% of successful cases and, among the residual leaks detected, none was larger than 2.5 mm.

There were 7 periprocedural major adverse events: 5 cases of cardiac tamponade [3 of them late (24h - 5 days after intervention); 4 of 5 were treated with pericardiocentesis, however the other required surgical drainage], the non-dedicated device embolization mentioned above, and a coronary air embolism without sequelae. Minor complications occurred in 4 patients (4.4%): one pericarditis (post-tamponade), one discrete pericardial effusion without clinical repercussions, one case of post-procedural pulmonary congestion and one arteriovenous fistula. After a median length of stay in hospital of two days, all the patients but 2 (one considering the assistant clinician's preferences, the other for presenting one ulcerated plaque in the aorta) were discharged with the prescription of acetylsalicylic acid and clopidogrel, without OAC.

Clinical follow-up was obtained in 97.8% of patients - 2 patients were lost to follow-up. After a period of 128.6 patient-years (median = 346 days and interquartile range of 195 to 985 days), there were three deaths unrelated to the procedure. There were two episodes of major bleeding: one of them in a patient with unsuccessful LAAC, which continued on warfarin therapy; the other was a gastrointestinal bleeding in a patient on dual antiplatelet aggregation therapy. Periprosthetic residual flow (all less than 2.5 mm) persisted in 5 of the 6 patients in whom they were originally detected, none of them with clinical consequences. No late development of residual flow was detected. In 2 patients, thrombus formation was detected over the device, both treated successfully after resuming OAC for three months. Only two patients (2.2%) had ischemic stroke at follow-up: one after six months, and the other 9 months after the intervention.

## Discussion

The basis for the hypothesis that systemic embolism can be prevented by closure of the LAA was the demonstration that, in patients with NVAF, more than 90% of atrial thrombi originate in this structure.^12^ After the initial experience with the PLAATO device^13^ and with the use of non-dedicated Amplatzer occluders,^14^ more than 3500 patients were included in 2 randomized and several observational studies with the Watchman device,^9,15-17^ whose results led to the approval of the device by the Food and Drug Administration (FDA) in 2015. Several unicenter and multicenter registries with the ACP device and its last generation, Amulet, were also published, the biggest of them including more than 1000 patients.^11,18-23^ Because of the favorable results of the intervention, the European Guidelines for the Management of Atrial Fibrillation validated the LAAC, in 2012, as a therapeutic strategy for patients with NVAF at a high stroke risk with a recommendation class IIb and a level of evidence B.^24^ Surprisingly, this level of recommendation did not evolve in the guidelines upgrade, published in 2016.^25^ The current Guidelines of the American College of Cardiology / American Heart Association / Heart Rhythm Society for the management of patients with atrial fibrillation, published in 2014,^6^ do not yet include recommendations on indications for LAAC. However, considering the technical developments of the procedure, the recent FDA approval of the WATCHMAN device and, especially, the last favorable results from the PROTECT AF trial, which showed a significant reduction in mortality compared with OAC in the late follow-up,^26^ the use of LAAC in clinical practice has expanded significantly in the USA, and it is anticipated that these guidelines recommendations will be updated soon.^27^ Published in 2016, and in accordance with this new body of information, the II Brazilian Guidelines for Atrial Fibrillation recognize LAAC as a valid alternative to OAC, with a class IIa recommendation, both for patients at high risk for thromboembolic phenomena and with contraindication for oral anticoagulants (level of evidence B), and for those with cardioembolic ischemic stroke despite correct use of oral anticoagulants (level of evidence C).^28^

One of the biggest limitations of the PROTECT-AF trial, a reference study on LAAC, was the unexpected complication rate of 7.7% associated with the implantation of the Watchman filter device.^9^ With the ACP device, national and international registries show that complication rates vary between 3.8% and 7.3%.^11,19,22^ Although within this range, the rate of complications in the Brazilian Registry is relatively high, probably as a reflex of the beginning of the learning curve of most operators with both prostheses. A review of the literature shows, however, that continued experience with the intervention decreases the complication rate of the procedure to as low as 2.8%.^17^

**Figure 5:**
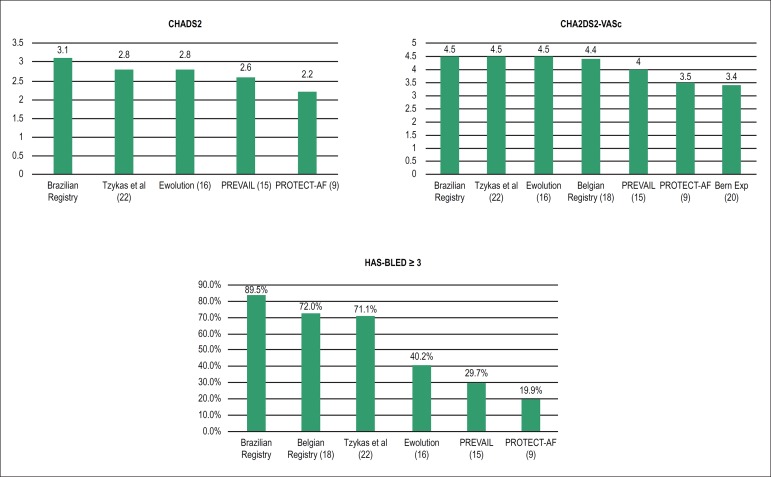
Comparison between mean CHADS_2_ (5a) and CHA_2_DS_2_-VASc (5b) scores and proportion of patients with HAS-BLED score ≥ 3 (5c) in the populations studied in the Brazilian Registry of Percutaneous Left Atrial Appendage Closure vs other registries and trials

The Brazilian Registry of Left Atrial Appendage Closure treated the population with the highest risk profile for systemic embolism and bleeding, compared to all registries and trials available in the literature. CHADS_2_ and CHA_2_DS_2_-VASc average scores of 3.1 and 4.5 are equal or higher than those related to the study populations in the PROTECT-AF,^9^ PREVAIL,^16^ Ewolution^17^ trials and in the multicenter experience with the ACP^23^ (2.2 and 3.5, 2.6 and 4.0, 2.8 and 4.5 and 2.8 and 4.5 respectively - Figure 5). Nonetheless, the annual stroke rate during the follow-up was notably low (1.7% - 2 events/128.6 patient-years, a reduction of 68.5% compared to the 5.4% annual rate estimated by the CHA_2_DS_2_-VASc score). This rate is between the 1.6% demonstrated in the meta-analysis, which includes the Watchman trials^29^ and the 1.8% demonstrated by Tzikas et al.^23^ with the ACP trial, and confirms the efficacy of the intervention in our population.

Due to the underutilization and to the discontinuity of treatment, both reaching rates of up to 40%,^29^ OAC reaches only a fraction of its therapeutic potential. For adherent patients, the risk of major bleeds remains significant. In spite of a best-use profile, the administration of NOACs is still associated with the occurrence of major bleeding in 2-3% of patients/year, even in those at low risk.^7^ The older the patient, the higher the rates and the severity of bleeding. A recent study with 32000 American veterans aged over 74 years and with AF treated with warfarin showed a hospitalization incidence due to traumatic intracranial hemorrhage of 4.8/1000 patient-years, and 6.2/1000 patient-years, when multiple events per patient are included.^30^ In this sense, the Brazilian Multicenter Registry showed that the bleeding rate was reduced by 77% compared to the expected rates based on the HAS-BLED score (1.7 versus 7.4 events/100 patient-years). This is especially significant considering that 83.5% of patients had a high bleeding risk with a HAS-BLED score ≥ 3 - also the worst risk profile compared to other studies available in the literature (Table 2 and Figure 5). If we consider only the patients effectively treated with LAAC, this rate is even lower, since one of the bleedings occurred in one of the patients whose intervention was unsuccessful, and this patient was treated with OAC.

Although thrombus formation at the atrial sides both of the Watchman device and of the ACP has been reported in 2 - 5% of cases, thromboembolic stroke rates secondary to this cause are very low (0.3 - 0.7%), and in general thrombus resolution is obtained after resuming OAC for short periods of time (< 3 months).^31^ This was also the case for the 2 patients in this Registry in which thrombus over the device was detected in the follow-up. Periprosthetic residual flow, found in 6 patients immediately after the intervention and which persisted in 5 of them at the follow-up, is also frequently described with both prostheses, but does not seem to have clinical significance if it is less than 5mm,^32,33^ which was also the case in all 6 patients.

The clinical benefits of LAAC increase when patients with higher CHA_2_DS_2_-VASc and HAS-BLED scores are treated, and they become more evident over time, due to the interruption of cumulative bleeding risk associated with continuous anticoagulation therapy.^34^ In addition to the reduction of objective stroke and bleeding rates, however, patients submitted to LAAC also experience a more subjective, but significant, quality of life improvement, especially due to the reduction of minor bleedings and to the lack of need for frequent monitoring, interactions with food and drugs and lifestyle restrictions associated with OACs.^35^ These factors, although less measurable, must also be taken into account when the risk-benefit ratio of the intervention is calculated.

## Conclusion

In conclusion, LAAC has proven to be effective in a real-world population with high-risk AF for reducing significantly the annual stroke and bleeding rates when compared to the expected rates based on CHA_2_DS_2_-VASc and HAS-BLED scores. The complication rates of the procedure must be weighed against the risks, discomforts and limitations associated with continuous and uninterrupted exposure to OAC.

### Limitations

This study has several limitations. As an inherent limitation to a non-randomized study, there is no control group, and the comparison of event rates was based on rates predicted by scores. As in every observational study, there may be flaws in patient selection. However, the Registry was designed in order to include all the patients who were candidate for the procedure (intention to treat), reflecting a real-world practice. Although the data have been prospectively collected, this is a retrospective analysis, without independent monitoring, or a core lab analyses. Especially due to reimbursement difficulties in Brazil, basically all centers included in this Registry are centers with low volume of LAAC and, thus, the learning curve of the operators is flattened, which has a direct impact on complication rates. The follow-up included more than 95% of patients treated, but not all of them. And, finally, all the data collected were spontaneously reported by investigators, without independent adjudication.
